# The Envy of Scholars: Applying the Lessons of the Framingham Heart Study to the Prevention of Chronic Kidney Disease

**DOI:** 10.5041/RMMJ.10214

**Published:** 2015-07-30

**Authors:** Walter G. Wasser, Amnon Gil, Karl L. Skorecki

**Affiliations:** 1 Division of Nephrology, Mayanei HaYeshua Medical Center, Bnei Brak, Israel; 2 Division of Nephrology, Rambam Health Care Campus, Haifa, Israel; 3 Division of Nephrology, Carmel Medical Center, Haifa, Israel; 4 Ruth & Bruce Rappaport Faculty of Medicine, Technion-Israel Institute of Technology, Haifa, Israel; 5 Director of Medical and Research Development, Rambam Health Care Campus, Haifa, Israel.

**Keywords:** ACE inhibitor, chronic kidney disease, FGF23, proteinuria, risk-factors

## Abstract

During the past 50 years, a dramatic reduction in the mortality rate associated with cardiovascular disease has occurred in the US and other countries. Statistical modeling has revealed that approximately half of this reduction is the result of risk factor mitigation. The successful identification of such risk factors was pioneered and has continued with the Framingham Heart Study, which began in 1949 as a project of the US National Heart Institute (now part of the National Heart, Lung, and Blood Institute). Decreases in total cholesterol, blood pressure, smoking, and physical inactivity account for 24%, 20%, 12%, and 5% reductions in the mortality rate, respectively. Nephrology was designated as a recognized medical professional specialty a few years later. Hemodialysis was first performed in 1943. The US Medicare End-Stage Renal Disease (ESRD) Program was established in 1972. The number of patients in the program increased from 5,000 in the first year to more than 500,000 in recent years. Only recently have efforts for risk factor identification, early diagnosis, and prevention of chronic kidney disease (CKD) been undertaken. By applying the approach of the Framingham Heart Study to address CKD risk factors, we hope to mirror the success of cardiology; we aim to prevent progression to ESRD and to avoid the cardiovascular complications associated with CKD. In this paper, we present conceptual examples of risk factor modification for CKD, in the setting of this historical framework.

Rav Dimi from the Babylonian Talmudic Academy of Nehardea said: “Jealousy between scholars increases wisdom.”

Babylonian Talmud, Tractate Bava Batra 21a

## INTRODUCTION: CONTRASTING CARDIOLOGY AND NEPHROLOGY

**Figure 1:**
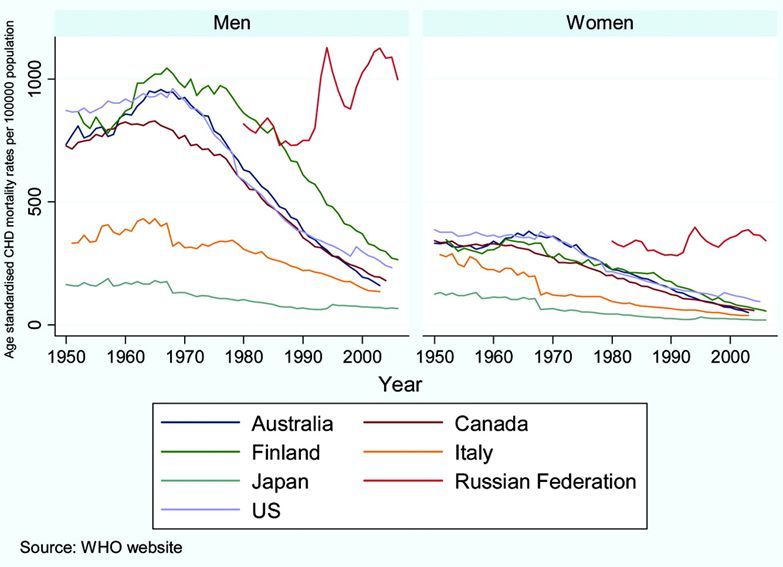
Global Age-standardized Coronary Heart Disease (CHD) Mortality Rates in Men and Women 45 to 74 Years of Age, Based on World Health Organization Statistics Copyright 2010, Wolters Kluwer Health, Inc. Used with permission from Cooney et al.^1^ Promotional and commercial use of the material in print, digital or mobile device format is prohibited without the permission from the publisher Wolters Kluwer Health.

Over the last half century, we have witnessed a global reduction in the coronary heart disease mortality rate by approximately 60% (Figure 1).^1^ Cardiovascular disease mortality rates in the US dramatically decreased from 805 deaths per 100,000 people in 1963 to 236 per 100,000 people in 2010.^2^ Before that time, the incidence of cardiovascular disease-related death was on the rise. Myocardial infarction and sudden death would occur without warning, striking down individuals in mid-life, during the peak of their productivity.^3^ In addition, the pathophysiology of these disorders was not understood.

It was in this context that the US National Heart Institute launched and co-ordinated the Framingham Heart Study in 1949. This study would become a cornerstone in cardiac epidemiology, heralding numerous follow-up studies in different constituencies and formats. The study, which began with 5,209 patients who were followed longitudinally, is still ongoing and has enrolled three generations of participants. The analysis gave rise to the concept of risk factors for coronary disease, including hypertension, high cholesterol, and smoking. This recognition led to vigorous risk-reduction campaigns.^4^–^6^

Models of the decrease in cardiac mortality from 1980 to 2000 found that risk factor reduction explained 44% of the reduction in cardiac death; treatment was responsible for an additional 47% reduction in mortality. Furthermore, reductions in total cholesterol, blood pressure, smoking, and physical inactivity accounted for 24%, 20%, 12%, and 5% reductions in the mortality rate, respectively.^7^

Cardiovascular risk prediction formulae, which are important for gauging individual cardiovascular risk, are also useful for understanding population-wide coronary disease risk.^1^,^8^ The Framingham risk estimation system, the most commonly used tool for this purpose, has been adjusted for use in various countries and was developed via cardiac epidemiological studies. Research is presently underway to incorporate sets of single-nucleotide polymorphisms (SNPs) obtained from genome-wide association studies (GWAS) to increase the accuracy of coronary heart disease risk determination.^9^

Paralleling these developments, clinicians who received scientific fellowship training in the laboratories of renal physiologists returned to their academic internal medicine departments to create divisions of nephrology.^10^ These departments supported active research on renal physiology while also providing clinical care to nephrology patients.^11^ The first kidney biopsies were performed in the 1950s. Although hemodialysis was first performed in 1943, it was typically performed outside of these departments because the procedure was viewed in many centers as academically unworthy.^10^ Kidney transplantation was first developed in 1963.^12^ The provision of dialysis therapy to people with kidney disease challenged the young specialty. Hemodialysis initially lacked specific funding, and committees such as the Admissions and Policies Committee of the Seattle Artificial Kidney Center at Swedish Hospital determined which patients would receive treatment.^13^ Such groups, consisting of seven citizens selected by The Kings County Medical Society, were formed to prevent doctors from needing to make these decisions regarding their own patients. Although the deliberations of the “God committee” were secret, a prominent article in *Life Magazine* detailing the thinking involved did emerge.^13^

The idea of federal funding for end-stage renal disease (ESRD) was debated among clinicians, and a vocal minority backed Boston nephrologist Dr Norman Levinsky who wrote in the influential *New England Journal of Medicine* in August 1964 that “both chronic dialysis and transplantation … are properly considered clinical experiments rather than established modes of treatment at this time.”^14^ Dramatically, in October, 1971, Shep Glazer, then Vice President of the National Association of Patients on Hemodialysis testified before the House Ways and Means Committee while being dialyzed. In 1972, congressional approval was attained to expand funding for the Medicare dialysis program; soon afterward, nearly every nephrology division embraced dialysis. The creation of the ESRD Program as part of the Medicare program for patients of any age who required dialysis tasked nephrologists with the substantial job of providing dialysis treatments, an endeavor that overwhelmed, hindered, and did not provide incentives for the performance of epidemiologic research for the identification and mitigation of risk factors in order to reduce the onset and progression of chronic kidney disease (CKD). Physiological research regarding the pathogenesis of chronic kidney disease led to new treatments for patients and a vital taxonomy of kidney diseases; however, it did not significantly influence the treatment of the majority of individuals with CKD. Over the next 60 years, the progressive advances in hemodialysis technologies did not affect the dialysis patient 5-year mortality that remained at ~50% (a mortality rate just slightly lower than that of lung cancer).^15^

Although the initial estimates of individuals who would require dialysis were low, the number of patients receiving dialysis treatment increased exponentially. From an initial 5,000 patients in 1972, the US ESRD program expanded more than 100-fold to 636,905 patients by 2012.^16^ Today, although 17,330 kidney transplants are performed annually, 81,981 patients remain on the active transplant waiting list, and numerous kidney transplantation candidates die while still on dialysis.^16^

Numerous pathophysiological studies, particularly those conducted by Drs Neal Bricker and Barry Brenner, have led directly to a paradigm shift in the treatment of CKD. Bricker proposed the “trade-off hypothesis,” in which he provided evidence that the production of hormonal factors in the setting of chronic renal failure was a homeostatic adaptation and not a consequence of a reduced glomerular filtration rate.^17^,^18^ As examples, he listed parathyroid hormone (PTH) and natriuretic factor. Bricker postulated that a circulating inhibitor of sodium transport alters the net movement of sodium from tubular fluid to the blood; recently this factor was purported to have been isolated.^19^

Brenner and colleagues showed that intraglomerular hypertension increases in residual nephrons following nephron loss. Systemic hypertension also increases intraglomerular pressure, which is modulated by the vascular tone of the pre- and post-glomerular arterioles, intraglomerular architecture, and hemodynamics. Elevated glomerular capillary pressure leads to an increased number of large non-selective pores on the glomerular capillary wall, which promotes proteinuria.^20^–^22^ Growth-promoting factors are released in the remnant glomeruli, and these factors produce excessive extracellular matrix in the mesangial area, obliterating the capillary lumen and creating typical sclerotic lesions. Nephron loss is increased, and this effect augments these processes in other glomeruli.^22^ Glomerular hypertrophy in remnant nephrons, compensatory to nephron loss, also contributes to glomerular sclerosis. The latter effect was reduced in a rat model of nephron loss without hypertrophy, compared with five-sixths of nephrectomized rats with a higher glomerular area, despite similar elevations in intraglomerular pressure.^23^ Brenner and colleagues showed that the inhibition of the vasoconstricting effect of angiotensin II via angiotensin-converting enzyme (ACE) inhibitors, which is most pronounced at the level of the post-glomerular arterioles, reduces intraglomerular hydraulic pressure. The effect of these agents on kidney injury progression supports the association between high glomerular pressure and sclerosis. In addition, angiotensin II inhibition reduces the synthesis of reactive oxygen species, inflammatory cytokines, cell adhesion molecules, and profibrotic molecules such as TGFβ.^24^

## KIDNEY DISEASE AS A PUBLIC HEALTH CONCERN

**Figure 2:**
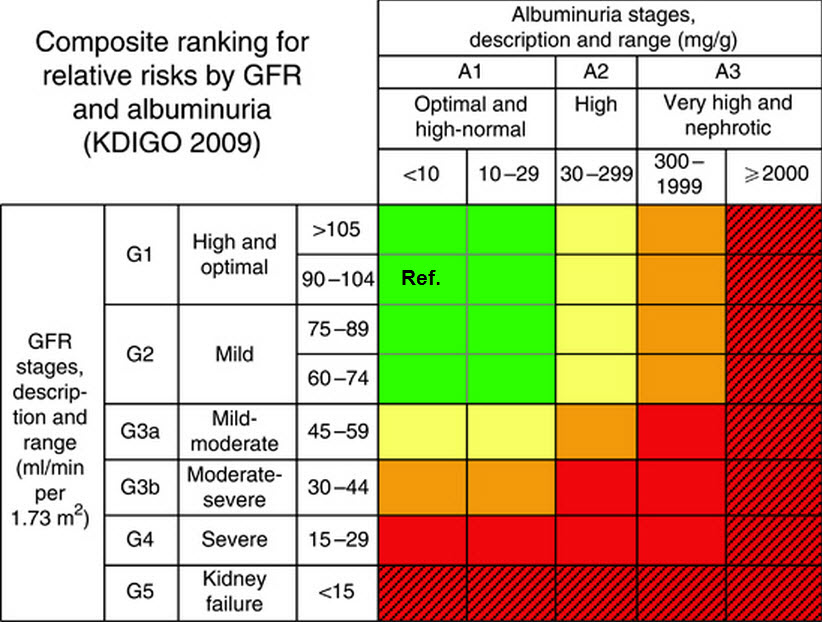
Composite Ranking for Relative Risks by Glomerular Filtration Rate (GFR) and Albuminuria Mortality is reported for general population cohorts assessing albuminuria as urine albumin-creatinine ratio (ACR). Kidney outcomes are reported for general population cohorts assessing albuminuria as either urine ACR or dipstick. Estimated glomerular filtration rate (eGFR) and albuminuria are expressed as categorical variables. All results are adjusted for covariates and compared with the reference cell (Ref). Each cell represents a pooled relative risk from a meta-analysis. Incidence rates per 1,000 person-years (calculated from the reference cells) are 7.0 for all-cause mortality, 4.5 for cardiovascular disease mortality, 0.04 for kidney failure, 0.98 for acute kidney injury (AKI), and 2.02 for kidney disease progression. Absolute risk can be computed by multiplying the relative risks in each cell by the incidence rate in the reference cell. See Levey et al.^25^ for full details. Colors on this heat map reflect the ranking of adjusted relative risk. The point estimates for each cell were ranked from 1 to 28 (the lowest RR having rank number 1, and the highest number 28). The categories with rank numbers 1–8 are green, rank numbers 9–14 are yellow, the rank numbers 15–21 are orange, and the rank numbers 22–28 are colored red. Color for twelve additional cells with diagonal hatch marks is extrapolated based on results from the meta-analysis of chronic kidney disease cohorts and represents the highest risk. The highest level of albuminuria is termed ‘nephrotic’ to correspond with nephrotic range albuminuria and is expressed here as 2000 mg/g. Figure and legend reprinted by permission from Macmillan Publishers Ltd: Kidney International,^25^ copyright 2011.

As the number of patients receiving dialysis care escalated, the potential associated costs began to alarm health care planners. In the words of the *Kidney Disease: Improving Global Outcomes (KDIGO) 2009 Conference Report*, the “rising prevalence, poor outcomes, and high costs of chronic kidney disease has led to its recognition as a public health threat.”^25^ Fundamentally, this recognition represented a paradigm shift for nephrologists and transformed kidney failure from a life-threatening condition that affected a few people (although these few required dialysis and transplantation) to a common condition that is the target of prevention, early detection, and management by non-nephrologist physicians and public health agencies.^26^ As a result, a quiet but significant revolution took place, beginning with the description of the model currently in use for CKD (Figure 2). This model spearheaded a redefinition of the diagnosis and treatment of CKD that relied on functional measures and the classification of kidney dysfunction via the degrees of albuminuria and nephron function loss (measured by estimated glomerular filtration rate, eGFR). The overwhelming numbers of patients with CKD has led nephrologists to follow cardiologists in using a Framingham-like model to identify the risk factors for CKD.

## CHRONIC KIDNEY DISEASE AS A MAJOR CARDIOVASCULAR RISK FACTOR

The association between ESRD and accelerated cardiovascular mortality has long been recognized.^27^ Mogensen first described microalbuminuria as a cardiovascular risk factor in people with diabetes.^28^ Bigazzi et al. showed the importance of microalbuminuria in predicting cardiovascular risk among people with hypertension.^29^ Recent meta-analyses have demonstrated the continuous associations among macroalbuminuria, microalbuminuria, coronary risk,^30^ and stroke.^31^ Other studies have shown that the use of renin–angiotensin–aldosterone system (RAAS) agents to decrease protein excretion can effectively reduce coronary risk.^32^–^36^

In line with these findings, screening 60% of the patients at highest risk has prevented virtually all forms of cardiovascular disease.^37^,^38^ However, elderly patients present “reverse metabolic syndrome”^39^,^40^ in which lipid levels and blood pressure are reduced, thereby making screening for coronary disease challenging. Detection techniques such as ultrasound measurement of the carotid intima-media thickness and CT scanning of the coronary arteries to show calcifications might accurately reveal subclinical atherosclerosis; however, these techniques are costly and therefore often unavailable. Estimated GFR and urinary albumin excretion might provide a cost-effective method to identify precisely the patients with cardiovascular disease.

**Figure 3:**
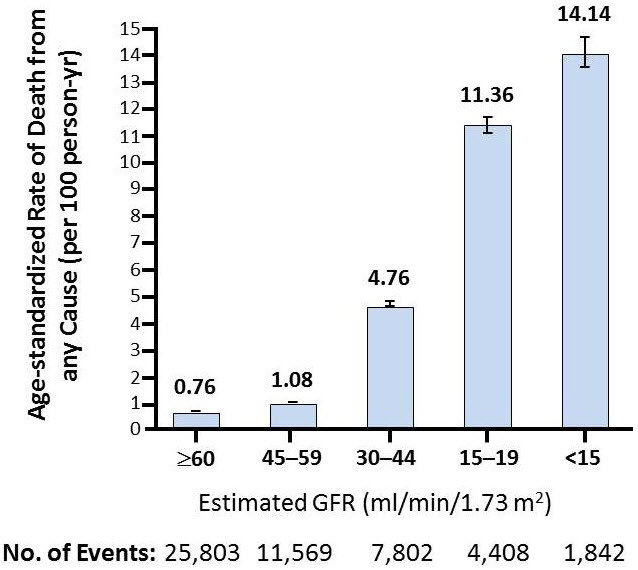
Death from Any Cause According to the Estimated GFR among 1,120,295 Ambulatory Adults Among a large, diverse population of adults from Kaiser Permanente Renal Registry, a reduced estimated GFR was associated with increased risks of death, cardiovascular events, and hospitalization that were independent of known risk factors, a history of cardiovascular disease, and the presence of documented proteinuria. These graded risks of adverse events rose sharply for subjects with an estimated GFR of <45 mL per minute per 1.73 m^2^ for each outcome examined both in the overall cohort and in subgroup analyses. Furthermore, in this cohort as a whole, the absolute rates of these outcomes were considerably higher than the risk of end-stage renal disease. From Go AS et al.^43^ Copyright ©2004, Massachusetts Medical Society. Reprinted with permission from Massachusetts Medical Society.

Small increases in serum creatinine are associated with cardiovascular events and mortality.^41^,^42^ Go and colleagues studied the health records of ~1.1 million adults in the Kaiser Permanente Renal Registry between 1996 and 2000 for >2 years and found an impressively graded association between estimated GFR and the risks for death, cardiovascular events, and hospitalization (Figure 3).^43^ Proteinuria was an independent risk factor for death. Chronic kidney disease, as determined via the combination of decreased renal function (estimated GFR) and markers of kidney damage (proteinuria), accurately predicted cardiac events and death more effectively than each individual risk factor alone.^44^–^47^ Foley and associates recently showed that the use of a “near-normal” estimated GFR cut-off of 94 mL/min and an albumin-creatinine ratio (ACR) of 9 mg/g, rather than the standard CKD thresholds, is highly sensitive and specific for selecting participants at risk of dying over the ensuing 9 years.^48^ Similarly high thresholds for estimated GFR without albuminuria successfully predicted cardiac death in two additional studies.^49^,^50^

Cardiovascular disease in the setting of CKD requires recognition and active treatment. Most patients with CKD succumb to cardiovascular disease rather than kidney disease. Recent meta-analyses^51^,^52^ and KDIGO guidelines^53^ recommend the use of statin therapy for patients with CKD but not those receiving dialysis.

## RISK FACTORS FOR PROGRESSIVE CKD

The effort to reduce CKD began with therapy for proteinuria and hypertension, which are recognized risk factors for CKD.^54^,^55^ The rate of progressive renal deterioration has a linear relationship with blood pressure treated by anti-hypertensive agents.^56^ Large controlled trials have documented the protein reduction properties of effective anti-hypertensive therapy.^57^–^59^ The success in implementing these therapies led to the identification of other CKD risk factors.

### Proteinuria

The relationship between reduced proteinuria and progressive renal disease was first demonstrated in the Modification of Diet in Renal Disease (MDRD) Study in 1995,^55^ which also showed that patients with high urinary protein excretion benefit more from ACE inhibitor-based therapies.^55^ The inhibition of the RAAS either via ACE inhibitors or angiotensin receptor blockers (ARBs) reduces proteinuria and progressive renal deterioration in excess of what would be expected based on the reduction of blood pressure alone with other non-RAAS agents.^60^ The ACE Inhibition in Progressive Renal Disease (AIPRD) study, a cumulative meta-analysis of 11 clinical trials including the Ramipril Efficacy in Nephropathy (REIN) study, found a strong correlation between proteinuria and the decline rate of glomerular filtration rate (GFR) in patients with CKD.^61^ Together, the MDRD and AIPRD studies revealed an impressive 40% reduction in the risk of doubling serum creatinine concentrations with ACE inhibitor treatment compared with other antihypertensive drugs in patients with CKD with protein levels >0.5 g per day.^62^ A similar effect was shown in patients with type 1 diabetes undergoing captopril treatment.^63^

The Reduction of End Points in NIDDM with the Angiotensin II Receptor Antagonist Losartan (RENAAL) study found that baseline albuminuria was the strongest predictor of the primary composite end-point of doubling of serum creatinine, end-stage renal disease (ESRD), or death in patients with type 2 diabetes mellitus (T2DM) with a serum creatinine level of 1.5–3.0 mg/dL.^34^ Overt proteinuria or microalbuminuria predicted kidney deterioration in a population with a high prevalence of CKD,^64^ individuals with hypertension and diabetes, and the general population.^29^,^65^,^66^ Significantly lower persistent microalbuminuria (6% in 3 years) was observed in patients with hypertension and T2DM with normal albuminuria treated by trandolapril compared to those receiving placebo (10%) or verapamil (11.9%).^67^ The anti-proteinuric and renal protection provided by ACE inhibitors was also observed in patients with diabetes and normal blood pressure but without microalbuminuria. The increase of albuminuria, even within the normal range, and the decrease of creatinine clearance were significantly lower in patients receiving enalapril compared with those receiving a placebo.^68^

The theoretical added therapeutic benefit of ARBs emanates from the blockage of angiotensin II to the angiotensin type 1 (ATI) receptor interactions as well as through enhanced angiotensin II binding to the vasodilatory angiotensin type 2 (ATII) receptors.^69^ Albuminuria decreased by 28% among losartan-treated patients over the first 6 months of the RENAAL study compared with a 4% increase among the placebo group. The decrease in albuminuria in the losartan group was associated with improved kidney function, going beyond the drug’s blood pressure-lowering effect.^34^ Changes in albuminuria showed an approximately linear relationship with the degree of long-term kidney protection. In particular, every 50% reduction in albuminuria was associated with a corresponding ~36% reduction in the risks of doubling of serum creatinine level, stage 5 CKD, or death. A similar effect was observed regarding other ARBs^32^,^70^ such as irbisartan. Treatment with ARBs has also been successful in patients with incipient diabetic nephropathy.^71^,^72^ The anti-albuminuric and renal protective effects of ARBs are similar, although slightly weaker, than the corresponding effects of the ACE inhibitors in early diabetic nephropathy.^73^

Despite the enormous progress that has been made in the treatment of progressive kidney disease via RAAS inhibition, the residual kidney risk after treatment with an ACE inhibitor or an ARB remains high and is associated with residual albuminuria.^74^,^75^ For example, in the RENAAL trial, losartan reduced the 3-year risk of doubling serum creatinine levels from 47% to 44%.^33^ In light of these high residual risk rates, recent reviews have examined various new strategies to enhance the effects of RAAS blockade.^69^,^76^ The RAAS is an endocrine cascade system that can be inhibited at many levels, but it can be compensated for at other levels with a clinical response known as “escape.”^69^,^76^

Clinical trials have examined the use of the combination of an ACE inhibitor and an ARB to prevent target organ damage.^77^ The Renal Outcomes with Telmisartan, Ramipril, or Both in People at High Vascular Risk (ONTARGET) trial compared the ACE inhibitor ramipril with the ARB telmisartan, alone and in combination, among patients at high risk for vascular disease.^78^,^79^ Although the achieved mean blood pressure was lower in patients who received telmisartan or both agents than in those who received ramipril alone, no difference was observed with regard to the primary outcomes among any of the groups, and more adverse outcomes were noted in the combination group. Importantly, this trial did not evaluate ARB and ACE inhibitor therapy in patients with advanced proteinuric renal disease. The VA Nephron-D Diabetes in Nephropathy Study (VA NEPHRON-D), a trial using a combination therapy (i.e. ACE inhibitor and ARB therapy versus ARB monotherapy) in patients with proteinuric diabetic nephropathy, was stopped because of the increased adverse events of hyperkalemia and acute kidney injury (AKI).^80^ The Aliskiren Trial in Type II Diabetes Using Cardiorenal Endpoints (ALTITUDE) randomly assigned 8,561 patients to aliskiren (300 mg daily) or a placebo as an adjunct to ACE/ARB monotherapy as an angiotensin receptor blocker. The trial was stopped prematurely because of adverse events (hyperkalemia and hypotension).^81^ Therefore, ACE inhibitors should not be used concomitantly with ARBs and renin inhibitors because of the increased risks for hypotension, hyperkalemia, and renal dysfunction.^82^

Many studies have attempted to achieve additional benefit from ACE inhibitors and other renin–angiotensin–aldosterone-blocking agents by increasing their dosages. This reasoning is based on the original observation that the optimal anti-proteinuric dose is not necessarily equal to the optimal antihypertensive dose. Many of these results have shown additional proteinuria reduction,^83^–^87^ whereas others have not.^88^–^90^ However, similar to the initial studies regarding combination therapy with renin–angiotensin–aldosterone-blocking agents, many of these high-dosage studies are also short-term examinations using blood pressure and albuminuria as outcome variables. These studies have not had sufficient power, and lack the duration needed to detect the safety signals and side effects rates that might emerge from end-point trials.^82^ Therefore, before ultrahigh RAAS-blocking agent dosing can be recommended as a renoprotective therapy, further study of these drugs with kidney and cardiovascular event data is needed.^82^

Optimization strategies for RAAS blockade have been suggested. First, a combination of sodium restriction and diuretic therapy is required to reach optimal RAAS inhibition in proteinuric patients.^91^–^96^ Second, hyperkalemia, which limits the use of RAAS agents, has recently received effective treatment with patiromer and sodium zirconium cyclosilicate among outpatients.^97^,^98^ These two novel drugs add to the pharmacopoeia that until recently was limited to sodium and calcium polystyrene sulfonate, which have adverse gastrointestinal effects.

Importantly, a few clinical caveats exist when treating proteinuria with RAAS inhibitors. First, greater initial decreases of renal function predict longer preservation of renal function.^99^ An initial loss of estimated GFR is not a concern unless it exceeds 30%, at which point diuretic-induced hypo-volemia and renal artery stenosis should be considered.^100^ In addition, the importance of monitoring urinary albumin decreases following RAAS blockade.^101^ If the urinary albumin–creatinine ratio is not lowered by ≥30% or to <300 mg/g despite a blood pressure lower than 130/80 combined with a low-sodium diet, then switching to another RAAS blocker or diltiazem should be considered.^79^,^102^ Greater reductions in proteinuria are seen with treatment using non-dihydropyridine calcium channel blockers (CCBs) than with dihydropyridine CCBs.^102^

### RAAS Inhibitors for Cardiovascular Protection

The ACE inhibitors reduce the rates of death, myocardial infarction, stroke, and heart failure among patients with heart failure,^103^ left ventricular dysfunction,^104^ previous vascular disease,^105^ and/or high-risk diabetes.^106^ The ARBs are an alternative for patients who cannot tolerate ACE inhibitors Although ACE inhibitors and ARBs have an additive effect, the more effective indication is to combine ACE inhibitor therapy with an aldosterone antagonist.

The MDRD study and other clinical interventions demonstrated strong interactions among proteinuria, hypoalbuminemia, blood pressure, CKD progression, and an increase in the inflammatory state.^55^,^107^ Furthermore, microalbuminuria is an independent risk factor for cardiovascular disease. In patients with T2DM, an albuminuria level of 20.1–30 mg/d was associated with a relative risk for cardiovascular disease of 9.8, and the relative risk for microalbuminuria was 12.4 compared with patients with albuminuria levels below 10 mg/d.^108^ The beneficial effect of albuminuria reduction for cardiovascular outcomes is likely associated with improvements in endothelial function in addition to the indirect effect mediated through the mitigation of renal dysfunction.

The ONTARGET trial assessed cardiovascular morbidity in patients with cardiovascular disease or high-risk diabetes but without significant albuminuria. Similar beneficial effects were observed regarding ARBs as ACE inhibitors for cardioprotection. However, the combination of these agents with ACE inhibitors was not associated with an increase in cardiac benefit, whereas adverse events were more common.^79^

### Hypertension

Hypertension is an uncontrolled and global public health challenge that is equally prevalent in developed and developing nations.^109^ In 2000, 25% of the world’s population had hypertension; however, approximately 29% (1.56 billion people) are expected to have hypertension by 2025. This increase has been ascribed to the massive “epidemiologic transition” of the developing world, with increasing proportions of elderly populations.^110^,^111^ Hypertension is the leading cause of cardiovascular morbidity and mortality and a major cause of CKD.

Perry et al. were one of the earliest groups to document carefully the association between increasing levels of systolic blood pressure, cardiovascular disease, and CKD risk.^112^ They described the direct association between increments in blood pressure elevation and the development of renal failure in 11,912 male veterans, 48% of whom were African-Americans, followed for 15 years at Veterans Administration Hypertension clinics during the mid-1970s. The risk ratios for a systolic blood pressure of 165–180 mmHg and of >180 mmHg were 2.8 and 7.6, respectively. Hospitalization for myocardial infarction doubled the risk for this disease; congestive heart failure increased the risk 5-fold and increased the rate of subsequent ESRD. The ESRD rate decreased by two-thirds among individuals whose systolic blood pressure fell by 20 mmHg.^112^ In addition, an increased risk of ESRD was associated with African ancestry (risk ratio=2.2).

The Multiple Risk Factor Intervention Trial (MRFIT) examined the development of cardiovascular complications in 12,000 men over 16 years and found that elevations in baseline systolic blood pressure were correlated with the development of ESRD, even within the high-normal and mild hypertensive ranges.^113^ This study also showed that effective blood pressure control stabilized or improved kidney function in Caucasians but not in African-Americans.^114^ In a 25-year observational study of 177,570 men and women, Hsu et al. demonstrated that small increases in systolic blood pressure within the pre-hypertensive and mild hypertensive ranges were correlated with increased CKD risk over time and an increase in the number of patients with ESRD.^115^ One risk factor for ESRD was high blood pressure (hazard ratio (HR) 2.94, 95% CI 2.21–3.92 for stage 2 hypertension; HR 2.33, 95% CI 1.78–3.05 for stage 1 hypertension; and HR 1.72, 95% CI 1.32–2.24 for pre-hypertension versus normal).

Forman and Brenner reviewed the evidence regarding a response to aggressive blood pressure reduction in “normotensive” individuals at high risk (diabetes, coronary artery disease, and cerebrovascular disease) and suggested maintaining a blood pressure below 120/80 in these patients.^116^ However, the clinical trials such as the ACCORD study,^117^ the Irbesartan Diabetic Nephropathy Trial (IDNT),^118^ and the International Verapamil SR-Trandolaptil Study (INVEST)^119^ found no benefit in bringing the blood below 130/80. Tight control of systolic blood pressure in the latter two studies did not yield improved cardiovascular outcomes and was in fact associated with an increase in all-cause mortality. These results have been summarized in other papers.^120^,^121^

Whether kidney and cardiovascular risks are lower in non-diabetic patients with CKD and blood pressures <130/80 compared with <140/90 remains unclear. This issue was examined by four randomized trials,^56^,^59^,^122^,^123^ two of which failed to show a significant benefit.^59^,^122^ However, the MDRD study showed that a reduction in blood pressure from <140/90 to <125/75 reduced the risk of kidney disease progression (HR 0.68) after 10 years of reduced blood pressure.^56^ Similarly, strict mean arterial blood pressure control in children below the 50th percentile for age versus a conventional therapy that corresponded to the 50th to 90th percentile for age led to decreased proteinuria and progression to ESRD (HR 0.65).^124^ The Cardio-Sis trial also demonstrated a benefit of blood pressure control in non-diabetic patients. Patients in the tight control group (<130 mmHg) developed less left ventricular hypertrophy (11% of 483 patients; odds ratio 0.63) and less frequently (4.8%) reached a composite cardiovascular end-point (HR 0.50) compared with patients under standard control (<140 mmHg; 17% and 9.4%, respectively).^123^

No evidence supports a preference for RAAS agents over other anti-hypertensive drugs among patients with CKD who present with hypertensive nephrosclerosis without proteinuria. The Antihypertensive and Lipid-Lowering Treatment to Prevent Heart Attack Trial (ALLHAT) did not find differences in the risk of exacerbated GFR or ESRD between patients given lisinopril, amlodipine, or chlorthalidone,^125^ even for the subgroup of patients with estimated GFRs <60 mL/min. Although proteinuria was not directly measured in these patients, it was not expected to be elevated.^126^

### Multidrug Remission CKD Clinic Protocols

Small annual differences in the rates of GFR decline can result in large differences regarding ESRD onset time.^127^ The goal of the RAAS-based individually tailored multidrug anti-proteinuric and antihypertensive treatments used over the last 15 years is to reduce proteinuria and the annual decline in eGFR.^128^ These protocols^129^ employ a combination therapy of ACE inhibitors and ARBs shown to reduce protein, kidney, and cardiovascular events more effectively than ACE inhibitors or ARB monotherapy.^127^,^129^,^130^ When proteinuria is minimal, a dual RAAS inhibitor is no more effective than a monotherapy (e.g. the ONTARGET Trial).^79^ The recent closure of the ALTITUDE and VA NEPHRON-D trials has placed the use of combination RAAS therapies on hold.^80^,^81^ A modified therapeutic strategy featuring a combination of lower-than-recommended doses of ACE inhibitors and ARB might block the RAAS system without excessive blood pressure reduction; moreover, the side effects of hyperkalemia and reduced kidney function are presently being investigated.^130^ The ongoing VALID trial, Preventing ESRD in Overt Nephropathy of Type 2 Diabetes Trial, is testing whether halved dosages of ACE inhibitor and ARB administered together compared with full doses of each agent alone result in a larger reduction in proteinuria and a delay in ESRD among approximately 100 individuals with type 2 diabetes over 3 years (ClinicalTrials.gov.NCT00494715). The trial will be completed in February 2016.

### Updating the “Trade-off” Hypothesis

#### Hyperparathyroidism

The progression of CKD and cardiovascular mortality have been directly correlated with changes in the levels of phosphate, calcium, PTH, vitamin D, and fibroblast growth factor 23 (FGF23).^131^ Laboratory studies in rats have demonstrated that PTH decreases glomerular filtration by decreasing the *K_f_* on the renal podocyte.^132^ The suppression of PTH via parathyroidectomy, calcimimetics (calcium-sensing receptor agonists), or dietary phosphate restriction attenuated the increase in serum creatinine in a rat remnant kidney model.^133^ Patients with CKD and secondary hyperparathyroidism have an increased mortality risk^134^ and a significantly shorter renal survival than those with CKD alone.^135^ Parathyroidectomy effectively reduces cardiovascular events and mortality in patients receiving hemodialysis with secondary hyperparathyroidism.^136^

#### Phosphate

The effect of phosphate on CKD progression might be directly mediated by changes in renal perfusion, calcifications, and intracellular calcium-phosphate concentrations, or through its indirect effects on PTH or calcium levels.^137^,^138^ The Irbesartan Diabetic Nephropathy Trial found that the risk of doubling serum creatinine levels, ESRD, or death^139^ was higher by a factor of 1.8 in hyperphosphatemic diabetics.^139^ The AASK analysis of African-American patients with hypertensive nephrosclerosis noted that phosphorus was directly associated with a renal composite consisting of 50%, 25 mL/min GFR decline, or ESRD.^140^ Additional studies have shown that increasing serum phosphate concentrations are correlated with progressive renal failure^141^–^144^ and that phosphate restriction^145^,^146^ and phosphate binders stabilize renal function.^147^,^148^ An analysis of the medical files of 40,538 outpatients receiving hemodialysis registered in the Patient Profile System of Fresenius Medical Care found that high phosphorus was correlated with increased relative risks of death (1.07, 1.25, 1.43, 1.67, and 2.02 for serum phosphorus levels of 5–6, 6–7, 7–8, 8–9, and >9 mg/dL, respectively). Higher adjusted calcium levels as well as moderate and severe hyperparathyroidism (PTH levels ≥600 pg/mL) were also associated with increased rates of death.^149^ High phosphate is also associated with increased mortality in patients with CKD.^141^,^149^,^150^ Serum phosphate levels within the normal range are associated with coronary artery calcification as determined by CT scanning in patients with stage 3 and 4 CKD with or without diabetes mellitus.^151^ The graded^152^,^153^ associations between serum phosphate levels of >3.5 mg/dL and coronary artery calcifications,^156^ cardiovascular disease, and mortality have also been extended to the general population.^155^–^159^

In studying the population disparities in mineral metabolism,^41^,^160^ African-American patients with CKD demonstrate marked deficiencies in serum 25-hydroxyvitamin D (25-OH vitamin D) and higher PTH levels than Caucasians.^161^–^163^ As these patients progress toward the need for dialysis, they show even more severe secondary hyperparathyroidism and 25-OH vitamin D deficiencies.^164^–^166^ This result was also shown by the multicenter Study to Evaluate Early Kidney Disease (SEEK) in 1,860 patients with early CKD, of whom 12% were African-American. African-Americans had significantly higher PTH, calcium, phosphorus, and bone-specific alkaline phosphatase levels. In addition, they had a 1.8-fold greater risk of elevated phosphate, a 2.7-fold greater risk of a 25-OH vitamin D deficiency <30 mg/mL, and a 4.7-fold greater risk of a severe 25-OH vitamin D deficiency. They also developed secondary hyperparathyroidism earlier in their CKD course at a GFR of 45–60 mL/min, whereas Caucasians generally developed hyperparathyroidism after their GFRs decreased to <30 mL/min.^167^ Gutierrez et al. showed that both healthy African-Americans and those with CKD had a fractional excretion of inorganic phosphate that was approximately 30% lower than that for Caucasians (*P*<0.001), and the fractional excretion of calcium in African-Americans was approximately 35% lower than in Caucasians. Both African-American and Caucasian patients with CKD had eGFRs between 15 and 60 mL/min, and they had similar PTH and FGF23 levels.^168^

Kestenbaum and colleagues described the results of a genome-wide association study (GWAS) that investigated common genetic variations associated with serum phosphorus concentrations in the general population.^169^ Seven loci were described, and one locus was found directly adjacent to SLC34A1, which encodes the kidney-specific type IIa sodium-phosphate cotransporter (NaPi2a). Another was located adjacent to the calcium-sensing receptor, and one was located close to the FGF23 receptor. The SLC34A1 was also one of the 13 loci identified by the CKDGen consortium, which performed a meta-analysis of the GWAS data in 67,093 individuals of European ancestry from 20 predominate population studies to identify new genetic susceptibility loci for reduced renal function.^170^ Although studied extensively in murine models where NaPi2a has been shown to serve as the central mediator of renal phosphate reabsorption,^171^,^172^ the role that this transporter plays in humans had been controversial until recently. Magen and associates recently described two siblings with autosomal recessive Fanconi’s syndrome and hypophosphatemic rickets who featured a 21-base-pair in-frame duplication on SLC34A1. Functional studies have shown a complete loss of function of the mutant cotransporter, its failure to reach the plasma membrane, and an impairment of renal phosphate reabsorption. This study provided the first evidence in humans of the critical role that NaPi2a plays in human renal phosphate handling.^173^ Subsequently, novel loss-of-function mutations in SLC34A1 were identified in members of families with idiopathic infantile hypercalcemia (IIH) not attributed to abnormalities in inactivation of vitamin D processsing, many with nephrocalcinosis.^174^,^175^

A gain-of-function mechanism might explain the hyperphosphatemia in patients with CKD, especially in light of its recent identification as a prominent CKD locus.^176^ People who tend toward lower fractional excretions of phosphate might exhibit increased levels of NaPi2a.^167^ The high and inducible levels of expression suggest that variant versions differ in folding and may trigger an “endoplasmic reticulum associated stress response (ERAD).”^177^

#### Vitamin D

The diminished production of 1,25-OH vitamin D in renal disease likely facilitates interstitial fibrosis by allowing fibroblasts to proliferate.^139^ Vitamin D has been shown to prevent glomerular disease in animal models,^178^,^179^ and its derivatives decrease urine albumin excretion as well as reducing serum creatinine and glomerulosclerosis in subtotally nephrectomized rats.^180^,^181^ In a retrospective analysis, patients with CKD who were treated with calcitriol showed a decreased rate of CKD progression.^182^ Increasing evidence has shown that the vitamin D analogue, paricalcitol, an inhibitor of the renin–angiotensin system,^183^ reduces urinary albumin. Three recent studies showed that paricalcitol reduces proteinuria in patients with CKD, including those presenting with diabetes.^184^–^186^ Paricalcitol reduced albuminuria and slowed the progression of kidney injury in laboratory animals.^187^,^188^ A recent double-blind, placebo-controlled study resulted in a 20% reduction in the urinary albumin-to-creatinine ratios (*P*=0.053) and a 28% reduction in the 24-hour urine albumin (*P*=0.009) of patients receiving 2 μg of paricalcitol for 24 weeks compared with those receiving a placebo.^189^

Decreased levels of 25-OH and 1,25-OH vitamin D are commonly observed in patients with CKD^190^ as well as associated with increased cardiovascular mortality.^164^,^191^ The treatment of CKD and ESRD populations using vitamin D compounds is associated with decreased mortality rates.^192^,^193^ However, a meta-analysis of 76 trials including 3,667 participants found that these compounds failed to reduce PTH levels or mortality rates consistently.^194^ Newer vitamin D compounds did decrease PTH levels (by 11 pmol/L); intravenous therapy was more effective than oral therapy, but mortality rates were not affected. Additional observational studies by the same group confirmed the reduction of serum PTH and the increase in calcium and phosphorus following treatment with vitamin D compounds but failed to show increased survival rates.^195^,^196^ These studies sparked a call for randomized controlled trials to establish a causal association between vitamin D supplementation and decreased CKD mortality.^197^

Although the decreased production of 1,25-OH vitamin D has traditionally been ascribed to decreased renal mass (which subsequently leads to elevated serum phosphate and the inhibition of reduced 25-OH D-1alpha-hydroxylase), these mechanisms fail to explain the decline in 1,25-OH vitamin D in patients with early CKD who still have sufficient kidney mass and normal serum phosphate levels.^198^

#### FGF23

**Figure 4:**
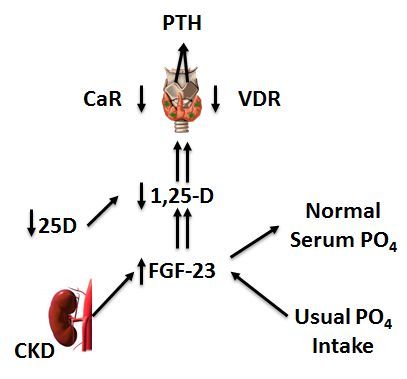
The Pathogenesis of Secondary Hyperparathyroidism (SHPT) in Chronic Kidney Disease (CKD) The new perspective of SHPT in CKD emphasizes the degree of phosphate intake relative to the degree of kidney dysfunction and de-emphasizes the need for overt hypophosphatemia or hypocalcemia. Early FGF23 excess may be a key upstream event of increasing PTH in CKD. Note also the early 1,25-D deficiency. Abbreviations: SHPT, secondary hyperparathyroidism; PTH, parathyroid hormone; CaR, calcium-sensing receptor; VDR, vitamin D receptor. Adapted from Figure 3 of Wahl and Wolf^201^ with the kind permission of the author and of Springer Science and Business Media (copyright 2012, Springer and Advances in Experimental Medicine and Biology).

Fibroblast growth factor 23 (FGF23), which was initially characterized in a study of rare inherited disorders associated with phosphate metabolism,^199^ regulates phosphate homeostasis and explains the decreases in 1,25-OH vitamin D in patients with early kidney disease. Its phosphaturic effect in the proximal tubules is accomplished through the down-regulation of sodium-phosphate co-transporters, and it decreases 1,25-OH vitamin D levels via the inhibition of 25-OHD-1-alphahydroxylase and the upregulation of the 25-OHD-24 hydroxylase pathway (Figure 4).^198^–^203^ Levels of FGF23 predict CKD progression from mild to moderate in patients of European ancestry,^142^ and they are among the strongest markers of CKD progression, with areas under the receiver-operating characteristic (ROC) curves of 0.84 for the C-terminal FGF23 and 0.81 for intact FGF23.^131^ Elevated FGF23 levels in patients with early CKD also predict early cardio-vascular events such as myocardial infarction and stroke as well as the need for coronary artery or carotid artery intervention, peripheral arterial amputation or intervention, and death.^204^ Elevated FGF23 levels are associated with increased mortality,^205^,^206^ vascular calcifications,^207^ left ventricular hypertrophy and mass index,^208^ and bone metabolism abnormalities in patients with ESRD.^209^ Strategies to reduce FGF23 in early-stage CKD patients include dietary phosphate restriction,^210^ the use of phosphate binders,^211^ the administration of niacin,^212^ and the restriction of administration of vitamin D-type drugs and favoring therapy with calcimimetics.^213^,^214^

The phosphate-regulating properties of FGF23 are mediated via FGFR1c, which requires alpha Klotho as a co-receptor. Its sites of action in the kidney are the subject of an active investigation and include the decreased expression of NaPi2a and NaPi2c.^215^ The recent demonstration of FGF23-mediated signaling in the distal convoluted tubule at the site of the alpha Klotho co-receptor adjacent to the NaPi2a-expressing proximal tubular cells likely represents FGF23 bioactivity through nephron-specific events that have yet to be elucidated.^216^ Saito et al. hypothesized that the initial aberration in this signaling pathway is the inappropriate upregulation of NaPi2a receptors;^217^ this theory is consistent with the association between the SLC34A1 locus and CKD described above.

An intriguing study recently found associations between FGF23 and body mass index (BMI), waist circumference, waist-to-hip ratio, serum lipids, and fat mass. In two cohorts of elderly European Caucasian participants, FGF23 was negatively associated with HDL and apolipoprotein A1 as well as positively associated with triglycerides. An increase of one standard deviation in the log-FGF23 levels was associated with a 7%–20% increase in BMI, waist circumference, and waist-to-hip ratio as well as a 7%–18% increase in trunk and total body fat mass as determined using whole-body dual X-ray absorptiometry. Levels of FGF23 were higher in participants with metabolic syndrome or at an increased risk of metabolic syndrome,^218^ indicating that FGF23 underlies cardiovascular risk via either phosphate or adverse lipid metabolism. The authors of that study cautioned against extending this association to African-American and Latino populations receiving dialysis; however, they emphasized that these populations have lower FGF23 levels^205^ and a dialysis survival advantage.^164^,^219^,^220^

Consistent experimental and human epidemiologic findings have suggested a need to test therapeutic approaches to lower phosphate levels in patients with CKD.^221^ Pilot studies of patients with stage 3 or 4 CKD suggest that phosphate binders, low phosphate diets, and vitamin B3 derivatives such as niacin and nicotinamide reduce phosphate absorption, serum phosphate, and FGF23. This novel therapeutic approach will be tested in the CKD Optimal Management with Binders and Nicotinamide (COMBINE) Study, with intermediate cardiovascular disease endpoints to include left ventricular hypertrophy (LVH), vascular calcification, and CKD progression.

### Obesity and Metabolic Syndrome

Obesity and associated metabolic syndrome, the results of Western dietary habits and sedentary lifestyles, exist at epidemic proportions in the US and are spreading worldwide.^222^ The continuous increase in obesity decreases life expectancy and general health.^223^ Obese participants are at greater risks for hypertension, insulin resistance and diabetes, hyperlipidemia, various cancers, and coronary vascular disease.^224^ Obesity and metabolic syndrome are also associated with pathologic renal changes and decreased renal function.^224^,^225^

Recently, the likely causes of the obesity epidemic were reviewed.^226^ Attempts to battle the problem have concentrated on decreasing fast food and trans fats intakes by providing nutritional information in stores and restaurants as well as reducing the consumption of soft drinks and high-fructose corn syrup.^222^ Numerous studies have associated increased trans fats intake with an increased risk of coronary disease.^227^–^229^ The intake of high-fructose corn syrup causes lipid abnormalities and hepatic insulin resistance.^230^ Epidemiologic studies show that the consumption of beverages containing a combination of sugars (including fructose) are associated with increases in body weight, metabolic syndrome, and cardiovascular disease.^231^ Similarly, the heightened use of artificial sweeteners is associated with obesity.^232^ Increasing evidence suggests that artificial sweeteners do not activate food reward pathways in the same manner as natural sweeteners,^233^ as demonstrated by the lack of the prolonged signal depression in the hypothalamus observed following glucose ingestion. Finally, sucrose ingestion, compared with saccharin ingestion, results in the greater activation of the higher gustatory areas such as the insula, orbitofrontal cortex, and the amygdala; this information might be useful for limiting energy intake.^233^–^235^

#### Obesity

The incidence of obesity, defined as a BMI of ≥30 kg/m^2^, has doubled since 1960. This condition affects one-third of the adult population in the US. The rise in overweight children from 6% to 19% over the past 25 years is even more alarming.^236^ Obesity is rapidly exceeding smoking as the leading cause of preventable death in the US.^224^ Eight studies have related excess body weight to the development of CKD and ESRD.^237^ An analysis of 320,252 members of the Kaiser Permanente Health System demonstrated that obesity is a risk factor for ESRD, with adjusted relative risks of 1.87, 3.57, 6.12, and 7.07 for those with BMIs of 25–29.9, 30–34.9, 35–39.9, and >40 kg/m^2^, respectively.^238^ Although the World Health Organization continues to use BMI to define obesity, the waist-to-hip ratio has been shown to predict more accurately the myocardial infarct risk worldwide.^239^ The link between obesity (defined by waist-to-hip ratio) and CKD has been reported.^240^ Even lean individuals with a high waist-to-hip ratio were at risk for developing microalbuminuria and a reduced estimated GFR.^241^ Obesity-related focal segmental glomerulosclerosis has also been described.^242^ The pathogenesis of this disorder is likely related to hyperfiltration, with increases in kidney mass and a glomerular hypertrophy effect. Hyperfiltration and increased filtration fraction are surrogate markers for elevated glomerular capillary pressures, which eventually result in obesity-associated glomerulosclerosis.^243^ Obesity-associated focal segmental glomerulosclerosis is associated with a lower rate of nephrotic syndrome and a more indolent course than idiopathic focal segmental glomerulosclerosis.^242^

Paradoxically, a strong association between increased body mass index (BMI) and lower mortality has been described in numerous studies of patients with stage 5 CKD undergoing maintenance hemodialysis with the benefits of a larger size extending into morbid obesity (BMI>35 kg/m^2^).^244^ This has been extended by two studies of patients with CKD where low BMI predicted greater mortality, whereas increased BMI was associated with greater survival even after adjustment for known confounding variables.^245^,^246^ The reasons for this association have not been determined.

#### Metabolic Syndrome

Associations between metabolic syndrome and both CKD and microalbuminuria have also been found in numerous studies, and Peralta et al. recently reviewed these relationships.^247^ After examining the data of 6,217 adults in the National Health and Nutrition Examination Survey III (NHANES), 24.7% of whom had metabolic syndrome, Chen et al. demonstrated graded relationships between the components of metabolic syndrome and the risks for CKD and microalbuminuria.^248^ Due to the cross-sectional nature of the study, determination of the temporal relationship between metabolic syndrome and CKD was not possible.^247^ The Atherosclerosis Risk in Communities Study (ARIC) examined more than 15,000 individuals and found that 21% (*n*=2,110) and 7% (*n*=691) developed metabolic syndrome and CKD, respectively, over a 9-year period; moreover, a similar graded relationship was found between the components of the syndrome and the risk for CKD. The odds ratio for the rate of CKD among participants with metabolic syndrome was 1.24 (95% CI 1.01–1.51).

Similarly, experimental hyperlipidemia models have demonstrated associations among progressive kidney damage, atherosclerosis, focal segmental glomerulosclerosis, and tubule-interstitial disease.^249^ Interestingly, a recent investigation of 19,246 participants in the southern US documented an association between a high saturated fat intake and albuminuria; however, no relationship was found with regard to decreased GFR.^250^ Moreover, an increased fructose intake (≥74 g/day) was implicated in obesity, metabolic syndrome, uric acid elevations, and hypertension; furthermore, it was a risk factor for kidney disease.^251^ High-fructose diets in animals led to renal hypertrophy, tubular cell proliferation, and injury. In a remnant kidney model, rats fed diets high in fructose developed metabolic syndrome and kidney disease progression.^252^ In humans, 6-week diets containing 25% fructose caused insulin resistance, visceral obesity, and abnormalities in serum lipids consistent with metabolic syndrome.^253^ The NHANES revealed an association between the ingestion of sugar-sweetened drinks and elevated uric acid levels; hypertension was also observed.^253^ Similarly, the Nurses’ Health Study found that ≥2 daily servings of artificially sweetened soda was independently associated with a ≥30% decline in estimated GFR over 11 years.^254^

A recent histopathologic study compared samples from 12 patients with metabolic syndrome undergoing nephrectomy for renal cancer with those from 12 controls.^255^ Samples from the patients with metabolic syndrome showed greater tubular atrophy, interstitial fibrosis, and arteriosclerosis as well as global and segmental glomerulosclerosis. These prominent interstitial changes led Saito et al. to postulate that the proximal tubular cell and, specifically, the multiligand megalin and cubilin receptors play a prominent role in the pathogenesis of this disorder.^217^

Welsh et al. recently engineered two mouse models lacking glomerular podocyte insulin receptors.^256^ Within 5 weeks, the animals began to show albuminuria and a shortening of the foot processes under electron microscopy. At 8 weeks, albuminuria, increased creatinine levels, the foci of segmental sclerosis, a thickening of the basement membranes, histologic evidence of apoptosis, and histopathologic features of diabetic nephropathy were observed, demonstrating the importance of podocyte insulin sensitivity in kidney function.^257^

According to the KDIGO guidelines, many patients with CKD should be treated with statins to prevent cardiovascular disease.^53^

#### Diabetes Mellitus

The importance of tight glycemic control to prevent kidney disease-related outcomes was recently demonstrated by the Diabetes Control and Complications Trial/Epidemiology of Diabetes Interventions and Complications Study (DCCT/ EDIC).^258^ The DCCT examined 1,441 participants with type 1 diabetes mellitus (1982–1993) assigned to intensive (median HgA1C 7.2%) versus conventional (9.1%) treatment for 6.5 years. Subsequently, participants were followed for >18 years in the observational EDIC. The intensive treatment used three or more daily insulin injections or insulin pump therapy guided by self-monitored glucose. During the DCCT, the intensive treatment reduced the rate of microalbuminuria (albumin excretion rate (AER) >40 mg/24 h) by 39% and that of macroalbuminuria (AER >300 mg/24 h) by 54% (24%–74%). During EDIC years 1–8, participants previously assigned to the DCCT intensive treatment experienced lower rates of microalbuminuria and macroalbuminuria, with risk reductions of 59% (30%–73%) and 84% (67%–92%), respectively. The beneficial effects of intensive therapy became evident at the end of the follow-up assessment, with reduced risks of impaired GFR (<60 mL/min) and hypertension of 50% (18%–69%) and 20% (6%–21%), respectively. The risk for retinopathy and neuropathy was also reduced, but not the risk for cardiovascular events.

With regard to type 2 diabetes, the kidney disease outcomes of the United Kingdom Prospective Diabetes Study (UKPDS),^259^ Kumamoto,^260^ Action in Diabetes and Vascular Disease Trial (ADVANCE),^261^ and Action to Control Cardiovascular Risk in Diabetes (ACCORD)^262^ trials are consistent with those of the DCCT for patients with type 1 diabetes.^258^ These analyses account for the relative differences in hemoglobin A1C achieved between treatment groups and the differences in study duration. A meta-analysis of type 2 diabetics^263^ featured 28 trials that included 34,912 participants with type 2 diabetes who were randomly assigned to an intensive glycemic control group (*n*=18,717) or a conventional glycemic control group (*n*=16,195). Targeting intensive glycemic control reduced the risk of microvascular complications (i.e. nephropathy and retinopathy) but increased the risks for hypoglycemia and serious adverse events. Tight glucose control confers long-term benefits regarding the prevention of progressive diabetic kidney disease.

Many individuals are not candidates for intensive glucose control in view of frequent episodes of hypoglycemia, impaired cognitive status, multiple comorbidities, and shortened life expectancies. Clinical guidelines have therefore recommended hemoglobin A1C targets as follows: specifically “individualized” care with A1C ~6.5% for healthy, young patients; <7% in older individuals or those with comorbid conditions, and <8% in older individuals with just a few years of life expectancy.^264^,^265^

### Other Risk Factors

Studies recently have identified uric acid,^266^–^268^ acidosis,^269^–^272^ and acute kidney injury^273^,^274^ as potentially modifiable risk factors for CKD.

## CONCLUSIONS

By nature, nephrologists are intelligent, creative, and competitive. As such, we are envious of the extraordinary success of our cardiology colleagues in the treatment of cardiovascular disease, and we wish to mirror their success. The aforementioned discussion of CKD risk factors makes the conceptual historical point of the importance of risk factor modification. Using this approach, in collaboration with our fellow clinicians, we can prolong the lives of individuals with kidney disease, target cardiovascular prevention, and decrease the number of patients referred for renal replacement therapy and kidney transplantation. Research presently under-way will target multiple novel pathways and identify multidrug approaches to accomplish these goals.^97^,^98^,^275^

The plateauing incidence of ESRD in the US over recent years indicates that these efforts have already shown success. The latest United States Renal Data System (USRDS) Annual Data Report from 2014 showed that the rate of ESRD has fallen from 368 cases per 1 million people in 2009 to 359 cases per 1 million people in 2012. The actual incidence has also fallen from 115,114 in 2009 to 114,813 in 2012.^16^ This marks the first time that the USRDS has reported a decrease in the number of new patients with ESRD since it began reporting in 1980. A decrease in incidence counts has also been reported for a number of other countries including Israel,^16^ perhaps reflecting attention to risk factor treatment.

## Abbreviations

ACCORD: Action to Control Cardiovascular Risk in Diabetes
ACR: albumin-creatinine ratio
ADVANCE: Action in Diabetes and Vascular Disease Trial
AKI: acute kidney injury
AIPRD: ACE Inhibition in Progressive Renal Disease
ALLHAT: Antihypertensive and Lipid Lowering Treatment to Prevent Heart Attack Trial
ALTITUDE: Aliskiren Trial in Type II Diabetes Using Cardiorenal Endpoints
ARB: angiotensin receptor blocker
ATI: angiotensin II type 1
ATII: angiotensin II type 2
CKD: chronic kidney disease
COMBINE: CKD Optimal Management with Binders and Nicotinamide
DCCT/EDIC: Diabetes Control and Complications Trial/Epidemiology of Diabetes Interventions and Complications
ESRD: end-stage renal disease
FGF23: fibroblast growth factor 23
GFR: glomerular filtration rate
IIH: idiopathic infantile hypercalcemia
KDIGO: Kidney Disease: Improving Global Outcomes
LVH: left ventricular hypertrophy
MDRD: Modification of Diet in Renal Disease
MRFIT: Multiple Risk Factor Intervention Trial
ONTARGET: Renal Outcomes with Telmisartan, Ramipril, or Both in People at High Vascular Risk Study
RAAS: renin–angiotensin–aldosterone system
NHANES: National Health and Nutrition Examination Survey III
REIN: Ramipril Efficacy in Nephropathy
RENAAL: Reduction of End Points in NIDDM with the Angiotensin II Receptor Antagonist Losartan
SEEK: Study to Evaluate Early Kidney Disease
T2DM: type 2 diabetes mellitus
UKPDS: The United Kingdom Prospective Diabetes Study
VALID: Preventing ESRD in Overt Nephropathy of Type 2 Diabetes
VA NEPHRON-D: Diabetes in Nephropathy Study, Combination Angiotensin Receptor Blocker and Angiotensin Converting Enzyme Inhibitor for Treatment of Diabetic Nephropathy.
